# Vitamin D and calcium supplementation in women undergoing pharmacological management for postmenopausal osteoporosis: a level I of evidence systematic review

**DOI:** 10.1186/s40001-025-02412-x

**Published:** 2025-03-14

**Authors:** Filippo Migliorini, Nicola Maffulli, Giorgia Colarossi, Amelia Filippelli, Michael Memminger, Valeria Conti

**Affiliations:** 1 Department of Orthopaedic, Trauma, and Reconstructive Surgery, RWTH University Hospital, RWTH University Medical Centre, Pauwelsstraße 30, 52074 Aachen, Germany; 2 Department of Orthopedics and Trauma Surgery, Academic Hospital of Bolzano (SABES-ASDAA), 39100 Bolzano, Italy; 3 Department of Life Sciences, Health, and Health Professions, Link Campus University, Via del Casale Di San Pio V, 00165 Rome, Italy; 4 Department of Medicine and Psychology, University La Sapienza, Rome, Italy; 5 School of Pharmacy and Bioengineering, Keele University Faculty of Medicine, ST4 7QB Stoke On Trent, England; 6 Barts and the London School of Medicine and Dentistry, Centre for Sports and Exercise Medicine, Queen Mary University of London, Mile End Hospital, E1 4DG London, England; 7 Department of Medicine, Academic Hospital of Würselen, Würselen, Aachen, Germany; 8 Clinical Pharmacology and Pharmacogenetics Unit, University Hospital “San Giovanni Di Dio E Ruggi d’Aragona”, 84131 Salerno, Italy

**Keywords:** Osteoporosis, Postmenopausal, Vitamin D, Calcium, Supplementation

## Abstract

The present systematic review investigates whether different doses of vitamin D and calcium supplementation in women with postmenopausal osteoporosis undergoing antiresorptive therapy have an association with BMD (spine, hip, femur neck), serum markers of osteoporosis (bone-ALP, NTX, CTX), the rate of pathological vertebral and non-vertebral fractures, adverse events, and mortality. This systematic review was conducted according to the PRISMA 2020 guidelines. PubMed, Google Scholar, Embase, and Scopus databases were accessed in September 2024. All randomised clinical trials (RCTs) comparing two or more treatments for postmenopausal osteoporosis supplemented with vitamin D and/or calcium were accessed. Only studies that indicated daily vitamin D and/or calcium supplementation doses were accessed. Data from 37 RCTs (43,397 patients) were retrieved. Patients received a mean of 833.6 ± 224.0 mg and 92.8 ± 228.7 UI of calcium and vitamin D supplementation, respectively. The mean length of the follow-up was 25.8 ± 13.3 months. The mean age of the patients was 66.4 ± 5.6 years, and the mean BMI was 25.2 ± 1.6 kg/m^2^. There was evidence of a statistically significant negative association between daily vitamin D supplementation and gastrointestinal adverse events (r = − 0.5; *P* = 0.02) and mortality (r = − 0.7; *P* = 0.03). No additional statistically significant associations were evidenced. In postmenopausal women who undergo antiresorptive treatment for osteoporosis, vitamin D was associated with a lower frequency of gastrointestinal adverse events and mortality. Calcium supplementation did not evidence an association with any of the endpoints of interest.

*Level of evidence* Level I, systematic review of RCTs.

## Introduction

Osteoporosis is a metabolic bone disease characterised by loss of bone mineral density (BMD) and bone mass and deterioration of bone microarchitecture, increasing the risk of fracture [1–3]. The prevalence of osteoporosis is very high, and the social and economic impact associated with osteoporosis-related fractures is particularly significant [4, 5]. The rate of bone loss increases with advancing age, especially in the first few menopausal years, constituting a major concern for women warranting the term postmenopausal osteoporosis (PMO) [6–9]. Since calcium and vitamin D play a synergistic role in preventing BMD loss and maintaining bone homeostasis [10–12], deficiency of these elements, especially in the elderly, is likely associated with alterations in bone remodelling and poor skeletal muscle health [13–15]. A diet rich in calcium and vitamin D appears to have a favourable impact on BMD. Since vitamin D status is critical for calcium absorption, the combined intake of these micronutrients could prevent hip fractures in postmenopausal women [3, 16, 17]. Pharmacological management of osteoporosis includes bisphosphonates, highly effective antiresorptive agents, as first-line therapy [18–20]. However, their effects vary among patients with a risk of treatment failure and adverse events [21, 22]. Given the chronic nature of osteoporosis, long-term treatment is necessary. Therefore, it becomes essential to identify therapeutic approaches that can complement drug therapy effectively and well-tolerated. Calcium and vitamin D supplements have been proposed as an anti-osteoporotic therapy recommended for their potential to reduce fracture rates in both institutionalised elderly and community-dwelling patients [23–25]. In PMO, combining vitamin D supplementation and bisphosphonates increases the efficacy of anti-osteoporotic treatment [26]. Vitamin D appears to play a role in enhancing the bisphosphonate tail effect on BMD after discontinuation of drug therapy [27]. However, although most randomised clinical trials (RCTs) have evaluated the efficacy and safety of antiresorptive drugs in patients receiving calcium and vitamin D supplementation, international guidelines now recommend individualising the use of these micronutrients according to risk factors for their insufficiency [28, 29]. Meta-analyses of RCTs reported only a weak effect on the occurrence of fractures and have drawn attention to possible side effects of calcium supplements previously ignored [30]. The role of calcium and vitamin D supplementation in the management of osteoporosis remains controversial, and data on the efficacy and safety effects in women with PMO undergoing antiresorptive treatment are lacking [31, 32].

The present systematic review investigates whether different doses of vitamin D and calcium supplementation in postmenopausal women undergoing antiresorptive therapy for osteoporosis are associated with BMD (spine, hip, femur, neck), serum markers of osteoporosis (bone-ALP, NTX, CTX), the rate of pathological fractures (vertebral and non-vertebral), adverse events, and mortality.

## Method

### Search strategy

This systematic review was conducted according to the PRISMA 2020 guidelines [33]. The PICOT algorithm was preliminarily established:

- P (Population): postmenopausal osteoporosis;
- I (Intervention): antiresorptive treatments;
- C (Comparison): vitamin D and calcium supplementation;
- O (Outcomes): BMD, serum markers, pathological fractures, adverse events, mortality;
- T (Type of study): RCT.

### Data source and extraction

Two authors (G.C. and M.M.) independently performed the literature search in September 2024. The following databases were accessed: PubMed, Google Scholar, Embase, and Scopus. The following keywords were used in combination: *osteoporosis, vitamin D, calcium, treatment, management, drug, pharmacology, pharmacological, medicament, mineral, density, bone, BMD, postmenopausal, spine, pathological, fragility, fractures, hip, vertebral, disability, adverse events*. The same authors independently performed the initial screening. The full text was accessed if the title and abstract matched the topic of interest. A cross reference of the bibliographies was also performed.

### Eligibility criteria

All randomised controlled trials (RCTs) comparing two or more treatments for postmenopausal osteoporosis supplemented with vitamin D and/or calcium were accessed. Only studies that stated daily vitamin D and/or calcium supplementation doses were accessed. According to the authors’ language capabilities, English, French, German, Italian, Portuguese and Spanish articles were eligible. Only RCTs level I evidence, according to the Oxford Centre of Evidence-Based Medicine [34], were considered. Articles including patients with glucocorticoid-induced osteoporosis were excluded. Studies conducted on patients with tumours and/or bone metastases and studies reporting data on patients with iatrogenic-induced menopausal and those on paediatric and/or adolescent patients were not included. Studies regarding selected patients undergoing immunosuppressive therapies or organ transplantation were not considered. Studies reporting data on combined therapy with multiple anti-osteoporotic or experimental drugs were also not included. Only articles reporting quantitative data under the outcomes of interest were eligible.

### Outcomes of interest

Two authors (F.M. and G.C.) independently examined the resulting articles for inclusion criteria. Study generalities (author, year, journal, length of the follow-up) and baseline demographic information were collected: the number of patients and relative mean age, mean bone mass index (BMI), mean BMD (spine, hip, femur neck), antiresorptive therapy. The outcome of interest was whether different doses of vitamin D and calcium supplementation have an association with BMD (spine, hip, femur neck), serum markers of osteoporosis (bone-ALP, NTX, CTX, PINP), the rate of pathological fractures (vertebral and non-vertebral), adverse events, and mortality.

### Methodology quality assessment

The risk of bias summary tool of the Review Manager Software (The Nordic Cochrane Collaboration, Copenhagen) was used to assess the methodological quality of the article included in the present systematic review. The following risks of bias were evaluated: selection, detection, attrition, and other sources of bias.

### Statistical analysis

The statistical analysis was performed by the main author (F.M.). The STATA Software/MP version 16 (StataCorporation, College Station, Texas, USA) was used for the statistical analyses. For descriptive statistics, the arithmetic mean and standard deviation were evaluated. The baseline comparability was assessed using the unpaired t-test, with *P* values > 0.1 considered satisfactory. A multivariate analysis diagnostic was used to analyse the association between the doses of vitamin D and calcium supplementation and the variables of BMD (spine, hip, and femur neck), serum markers of osteoporosis (bone-ALP, NTX, CTX), the rate of pathological fractures, adverse events, and death. Within studies, data concerning control groups or treatment arms which did not meet the inclusion criteria were not included in the statistical analyses. The Pearson product-moment correlation coefficient (*r*) was used. The linear regressions were evaluated according to the Cauchy–Schwarz inequality: + 1 (positive linear correlation) and − 1 (negative linear correlation). Values of 0.1 <|r|< 0.3, 0.3 <|r|< 0.5, and |r|> 0.5 were considered to have weak, moderate, and strong correlations, respectively. Overall significance was evaluated through the χ2 test. Values of *P* < 0.05 were considered statistically significant.

## Results

### Search result

**Fig. 1:**
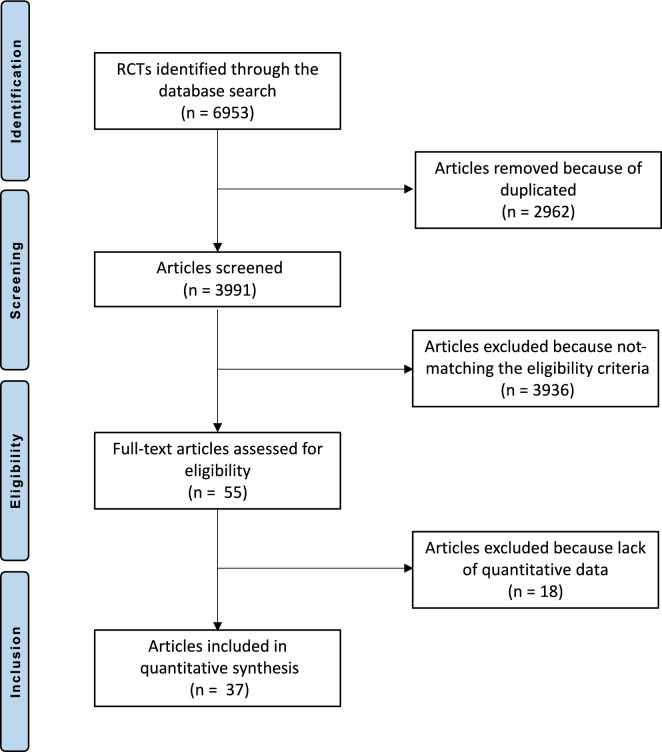
Flowchart of the literature search

A total of 6953 articles were identified from the four searched databases. Of them, 2962 studies were excluded as duplicates. An additional 3936 studies were excluded for the following reasons: not matching the topic (*N* = 1918), not reporting the exact amount of daily vitamin D and/or calcium (*N* = 1705), poor level of evidence (*N* = 149), referring to glucocorticoid-induced osteoporosis (*N* = 95), including patients with combined therapy with multiple anti-osteoporotic or experimental drugs (*N* = 21) language limitation (*N* = 19), including patients undergoing immunosuppressive therapies or organs transplantation (*N* = 13), including paediatric and/or adolescent patients with iatrogenic-induced menopausal (*N* = 9), including patients with tumours and/or bone metastases (*N* = 7). A further 18 articles were not eligible as they did not report quantitative data on the outcomes of interest. Finally, 37 RCTs were included in the present study (Fig. 1).

### Methodological quality assessment

**Fig. 2:**
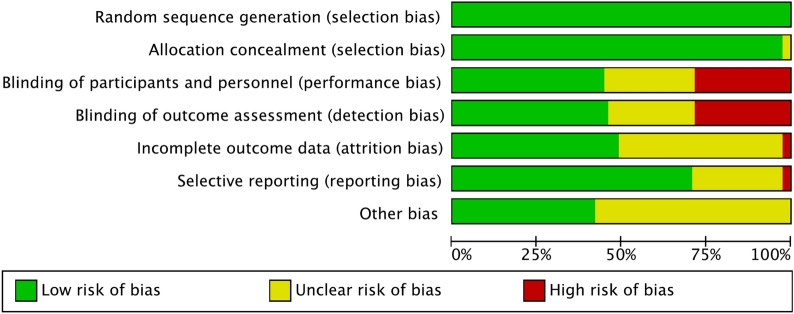
Methodological quality assessment

The risk of bias summary evidenced the strengths of the present study. First, the choice to include only RCTs reflected the low risk of selection bias. In addition, most patients and assessors were blinded, which resulted in a moderate–low risk of detection and performance bias. The high quality of the included studies also showed a low risk of attrition and reporting bias. In conclusion, the methodological assessment reported an overall low bias risk, leading to a very good methodological assessment (Fig. 2).

### Patient demographics

Data from 43,397 patients were retrieved. Patients received a mean of 833.6 ± 224.0 mg and 92.8 ± 228.7 UI of calcium and vitamin D supplementation, respectively. The mean length of the follow-up was 25.8 ± 13.3 months. The mean age of the patients was 66.4 ± 5.6 years, and the mean BMI was 25.2 ± 1.6 kg/m^2^. Study characteristics and patient data at baseline are shown in detail in Table 1.

**Table 1:** Study characteristics and patient data at baseline of the included studies

Author, year	Journal	Mean follow-up (*months*)	Mean calcium daily supplement (*mg*)	Mean vit D daily supplement (*UI*)	Treatment	Samples (*n*)	Mean age	Mean BMI (*kg/m2*)
Anastasilakis et al. [35]	*Osteoporos Int*	12	1000	800	Denosumab	32	63	28.80
					Zoledronate	26	63	28.70
Atmaca et al. [36]	*Adv Ther*	12	600	400	Alendronate	16	66	
					Risedronate	14	66	
Bai et al. [37]	*J Int Med Res*	24	600	400	Zoledronate	242	57	23.44
					Placebo	241	57	23.73
Body et al. [38]	*J Clin Endocrinol Metab*	14	1000	400–1200	Alendronate	73	65	24.40
					Teriparatide	73	66	23.90
Bone et al. [39]	*J Clin Endocrinol Metab*	24	813		Alendronate	86	71	
			880		Alendronate	89	70	
			831		Alendronate	93	71	
			900		Placebo	91	71	
Brumsen et al. [40]	*J Bone Min Res*	60	500	400	Pamidronate	26	66	
					Placebo	27	64	
Chesnut et al. [41]	*J Bone Min Res*	36	500	400	Ibandronate	977	69	26.20
					Ibandronate	977	69	26.20
					Placebo	975	69	26.20
Chung et al. [42]	*Calcif Tissue Int*	6	500	125	Ibandronate/risedronate	176	61	23.30
					Risedronate/ibandronate	176	62	23.40
Clemmesen et al. [43]	*Osteoporos Int*	36	1000		Risedronate	44	67	25.50
					Risedronate/placebo	44	68	24.40
					Placebo	44	70	25.10
Cummings et al. [44]	*JAMA*	48	634		Alendronate	2214	68	24.90
			638		Placebo	2218	68	25.00
Cummings et al. [45]	*New England J Med*	36	1000	400–800	Denosumab	3902	72	26.00
					Placebo	3906	72	26.00
Delmas et al. [46]	*J Clin Endocrinol Metab*	48	500	400–600	Raloxifene	2259	66	25.30
					Raloxifene	2277	66	25.20
					Placebo	2292	67	25.30
Ettinger et al. [47]	*JAMA*	36	500	400–600	Raloxifene	2259	67	
					Raloxifene	2277		
					Placebo	2292		
Fogelman et al. [48]	*J Clin Endocrinol Metab*	24	1000		Risedronate	184	65	24.80
					Risedronate	177	65	24.80
					Placebo	180	64	25.50
Gonnelli et al. [49]	*Bone*	12	841	400	Zoledronate	30	66	26.10
			870		Ibandronate	30	67	25.70
Greenspan et al. [50]	*JAMA*	24	807	163	Zoledronate	89	85	28.20
			763	168	Placebo	92	86	26.90
Grey et al. [51]	*J Clin Endocrinol Metab*	24	935		Zoledronate	25	62	
			916		Placebo	25	65	
Grey et al. [52]	*J Clin Endocrinol Metab*	12	960		Zoledronate	43	64	
			880		Zoledronate	43	66	
			850		Zoledronate	43	66	
			950		Placebo	43	65	
Guanabens et al. [53]	*Hepatology*	24	1000		Ibandronate	14	65	26.60
					Alendronate	19	63	26.60
Harris et al. [54]	*Am J Med*	48	500		Phosphate–etidronate	63		
					Placebo–etidronate	65		
					Phosphate–placebo	62		
					Placebo	63		
Harris et al. [55]	*JAMA*	36	1000	500	Risedronate	817	69	26.60
					Risedronate	821	69	26.60
					Placebo	820	68	26.50
Iwamoto et al. [56]	*J Orthop Sci*	24	800	400	Etidronate	25	64	21.20
					Menatetrenone	23	65	20.60
					Control (calcium lactate)	24	66	20.90
Liberman et al. [57]	*New England J Med*	36	500		Alendronate	175	64	24.20
					Alendronate	175		
					Alendronate	175		
					Placebo	355	64	24.10
Lufkin et al. [58]	*J Bone Min Res*	12			Raloxifene	48	67	24.80
					Raloxifene	47	67	26.20
			750	400	Calcium/ Vit D	48	68	25.30
McClung et al. [59]	*New England J Med*	12	1000	800	Romosozumab	44	67	
					Romosozumab	46	67	
					Romosozumab	49	67	
					Romosozumab	52	67	
					Romosozumab	53	67	
					Alendronate	47	67	
					Teriparatide	46	67	
					Placebo	47	67	
McClung et al. [60]	*J Bone Min Res*	12	1000	800	Denosumab	127	67	
					Placebo	131	67	
Meunier et al. [61]	*New England J Med*	36	1000	400–800	Strontium ranelate	719	69	26.20
					Placebo	723	69	26.20
Meunier et al. [62]	*Osteoporos Int*	12	1000	400–800	Strontium ranelate	221	72	
					Strontium ranelate	434	72	
					Placebo	225	72	
Miller et al. [63]	*J Clin Endocrinol Metab*	12	1000	800	Denosumab	321	69	24.30
					Zoledronate	322	70	24.30
Mortensen et al. [64]	*J Clin Endocrinol Metab*	36	937		Risedronate	37	52	
			1057		Risedronate	38	51	
			936		Placebo	36	51	
Neer et al. [65]	*New England J Med*	24	1000	400–1200	Teriparatide	444	69	
					Teriparatide	434	70	
					Placebo	448	69	
Paggiosi et al. [66]	*Osteoporos Int*	24	1200	800	Alendronate	57	68	25.90
					Ibandronate	58	67	26.40
					Risedronate	57	67	26.80
					Control	226	38	25.10
Peretz et al. [67]	*Maturitas*	24	500	400	Alendronate	18	68	
					Pamidronate	21	70	
Recknor et al. [68]	*Obstet Gynecol*	12	500	800	Denosumab	417	67	25.50
					Ibandronate	416	66	25.10
Reginster et al. [69]	*Osteoporos Int*	36	1000	500	Risedronate	410	71	
					Risedronate	408	71	
					Placebo	408	71	
Sanad et al. [70]	*Climacteric*	12	1500	400	Raloxifene	35	63	26.50
					Alendronate	31	62	25.80
					Raloxifene/ alendronate	32	63	26.30
Tucci et al. [71]	*Am J Med*	36	500		Alendronate	98	67	23.90
					Alendronate	94	64	23.30
					Alendronate	94	64	23.70
					Placebo	192	64	23.80

### Outcomes of interest

There was evidence of a statistically significant negative association between daily vitamin D supplementation and gastrointestinal adverse events (r = − 0.5; *P* = 0.02) and mortality (r = − 0.7; *P* = 0.03). No additional statistically significant associations were evidenced (Table 2).

**Table 2:** Results of the multivariate analyses

Endpoints	Calcium daily supplement (*mg*)		Vit D daily supplement (*UI*)	
	*r*	*P*	*r*	*P*
BMD spine	0,0	0,9	0,0	0,9
BMD hip	0,1	0,4	0,3	0,3
BMD femur neck	− 0,1	0,6	0,0	0,9
Serum bone-ALP	0,5	0,08	− 0,3	0,5
Serum NTX	− 0,2	0,5	0,1	0,8
Serum CTX	0,0	0,9	1,0	0,2
Pathological fracture of the hip	− 0,2	0,3	− 0,3	0,1
Any non-vertebral fracture (hip, ribs, wrist)	− 0,3	0,05	− 0,1	0,7
Pathological fracture of the spine	− 0,1	0,6	− 0,2	0,3
Adverse events	− 0,1	0,4	− 0,3	0,2
Serious adverse events	0,1	0,6	− 0,4	0,08
Gastrointestinal adverse events	− 0,2	0,3	− 0,5	0,02
Musculoskeletal adverse events	0,2	0,3	− 0,3	0,5
Mortality	0,2	0,6	− 0,7	0,03

## Discussion

According to the published level I of evidence articles, in postmenopausal women who undergo antiresorptive treatment for osteoporosis, vitamin D was associated with a lower frequency of gastrointestinal adverse events and mortality. Calcium supplementation did not evidence an association with any of the endpoints of interest.

Poor vitamin D status, identified by low serum levels of 25-hydroxyvitamin D (25(OH)D), has been associated with poor skeletal muscle health. Therefore, vitamin D, like calcium, has long been identified as a key element in preventing and treating bone loss and bone diseases such as osteoporosis [72]. A diet rich in calcium and vitamin D appears to have a favourable impact on BMD. Since vitamin D status is critical for calcium absorption, combining these micronutrients could prevent hip fractures in postmenopausal women [16]. However, the data available to date are conflicting, partly because of the heterogeneity of the studies regarding sample size and length of follow-up. In addition, one of the most debated issues concerns the variability of vitamin D dosages used. Some studies may not have revealed a significant effect, having probably used inadequate doses not commensurate with patients' needs. On the other hand, excess vitamin D might have less than positive effects, leading to reduced BMD and increased risk of fractures [16].

The present study found a statistically significant negative association between daily vitamin D supplementation and mortality. This is an important finding because the strong association between osteoporosis and fracture risk, especially in older adults, consequently increases patients’ morbidity and mortality [73]. Some observational studies have shown a possible association between low vitamin D status and increased mortality [74, 75]. However, this association may be nonlinear and appears lost at serum 25(OH)D concentrations above 87.5 nmol/L [76]. Vitamin D might significantly improve the survival of elderly subjects living in institutional care. Notably, this finding was independent of the baseline vitamin D status [77]. In postmenopausal women with osteoporosis, vitamin D supplements could be associated with decreased mortality [78]. However, several randomised controlled trials (RCTs) have reported only a trend toward reduced mortality without reaching statistical significance [78, 79]. The influencing factors are undoubtedly multiple, including the variable age of study participants and supplement dosage, so the relationship linking them to total mortality rates remains to be clarified. Given the strong interaction between calcium and vitamin D, a major concern is whether the beneficial effects on improved skeletal health attributed to vitamin D may result from concomitant calcium supplementation [80]. LaCroix et al. [81] performed a thorough analysis to evaluate the effects of combined supplementation of vitamin D and calcium in 36,282 postmenopausal women aged 51–82 years already enrolled in the “Women's Health Initiative (WHI) trial of CaD”, which had shown non-significant reductions in all-cause mortality [79]. Calcium/vitamin D supplementation reduced the risk of all-cause, cardiovascular, and cancer death in women younger than 70. In contrast, in older women, this combined treatment was only associated with a reduction in cancer mortality [81]. It is essential to monitor treatment adherence, as age did not influence the effects on mortality. Still, calcium and vitamin D supplements reduced all-cause mortality rates in women who adhered to this treatment. In this large RCT, as in other studies, the effects of vitamin D could not be distinguished from those of calcium, and notably, fixed dosages of 1000 mg of calcium carbonate and 400 IU of vitamin D3 were used. Based on this evidence, the results of WHI CaD appear to be inconclusive [81], and whether vitamin D given as monotherapy or combined with calcium may be able to reduce all-cause mortality remains an open question.

The present study demonstrates that, in contrast to findings related to vitamin D, the use of calcium supplements was not associated with either mortality or the other endpoints evaluated. Indeed, beneficial effects were found in mixed populations, including women with postmenopausal osteoporosis, subjects receiving combined vitamin D and calcium supplementation and those treated with vitamin D only [82–85]. A recent meta-analysis suggested that vitamin D supplementation between 700 and 800 IU/d (but not at lower doses) should reduce the risk of hip and non-vertebral fractures by about 25% in subjects aged 60. However, the authors did not define the role (if any) of concomitant calcium supplementation [83].

Another question is the safety profile of supplementation. The present systematic review found no association between calcium supplementation and side effects. In contrast, the use of vitamin D was associated with a lower frequency of gastrointestinal toxicity. The risk of kidney stones is common in patients taking calcium and vitamin D supplements simultaneously, while gastrointestinal side effects have been reported in patients taking calcium [83]. While vitamin D supplementation may reduce cardiovascular risk, calcium supplementation may increase it [86]. Calcium-related gastrointestinal toxicity, which is very common, is associated with an unfavourable risk–benefit profile that often leads to poor long-term therapeutic adherence. As a result, some authors suggest that calcium supplementation should not be recommended [87].

This study has some limitations. Variability in the mean follow-up (6 to 48 months) was evident. A shorter follow-up might reduce the efficacy of the present research in identifying the rate of pathologic fractures and their association with vitamin D and calcium doses. Another limitation is that in all included studies, vitamin D was taken together with calcium, so it is not possible to assess clearly whether the association between vitamin D supplementation and reduced mortality rate would have been found in the absence of calcium supplementation. On the other hand, because calcium and vitamin D play a synergistic role in preventing BMD loss and maintaining homeostasis and bone health, most osteoporotic patients use these supplements concomitantly.

## Conclusion

In postmenopausal women receiving antiresorptive treatment for osteoporosis, vitamin D was associated with a lower frequency of gastrointestinal adverse events and mortality. Calcium supplementation showed no association with any of the endpoints of interest. Since calcium absorption depends on vitamin D status and given the favourable benefit/risk profile associated with vitamin D supplementation, vitamin D as monotherapy or calcium co-administration appears superior to calcium supplements alone.

## Data Availability

No datasets were generated or analysed during the current study.
